# Virtual Reality—A Supplement to Posturography or a Novel Balance Assessment Tool?

**DOI:** 10.3390/s22207904

**Published:** 2022-10-17

**Authors:** Oskar Rosiak, Anna Puzio, Dorota Kaminska, Grzegorz Zwolinski, Magdalena Jozefowicz-Korczynska

**Affiliations:** 1 Balance Disorders Unit, Department of Otolaryngology, Medical University of Lodz, The Norbert Barlicki Memorial Teaching Hospital, 90-153 Lodz, Poland; 2 Institute of Mechatronics and Information Systems, Lodz University of Technology, 90-924 Lodz, Poland

**Keywords:** virtual reality, posturography, balance assessment, posture, head mounted displays

## Abstract

Virtual reality (VR) is a well-established technology in medicine. Head-mounted displays (HMDs) have made VR more accessible in many branches of medical research. However, its application in balance evaluation has been vague, and comprehensive literature on possible applications of VR in posture measurement is scarce. The aim of this review is to conduct a literature search on the application of immersive VR delivered using a head-mounted display in posturographic measurements. A systematic search of two databases, PubMed and Scopus, using the keywords “virtual reality” and “posturography,” was performed following PRISMA guidelines for systematic reviews. Initial search results returned 89 non-duplicate records. Two reviewers independently screened the abstracts. Sixteen papers fulfilled the inclusion criteria and none of the exclusion criteria and were selected for complete text retrieval. An additional 16 records were identified from citation searching. Ultimately, 21 studies were included in this review. virtual reality is often used as additional visual stimuli in static and dynamic posturography evaluation. Only one study has attempted to evaluate a VR environment in a head-mounted display as an independent method in the assessment of posture. Further research should be conducted to assess HMD VR as a standalone posturography replacement.

## 1. Introduction

The human balance system is one of the most complex neural connections in the central nervous system. The input information is not only limited to the labyrinth, but also to visual and somatosensory information from joints and tactile information [1,2]. Dysfunction of any elements that comprise this system may lead to a perception of instability or a false sense of motion, otherwise described as vertigo and dizziness. A decline in vision function [3], proprioception, or the inner ear related to aging can lead to balance disorders [4] and result in falls, which are a significant cause of morbidity and mortality in older people [5]. 

To measure complaints related to vertigo and dizziness, a variety of subjective methods were developed that employ clinical questionnaires [6]. Objective measurements of the balance system focus on assessing oculomotor and vestibular function using videonystagmography, and postural evaluation. Static posturography (SP), which measures the displacement of the center of pressure (COP) on a two-dimensional plane, has been the centerpiece of research on human posture for many years. However, traditional force-plate sensors are limited to quiet stance posture studies, and measurements on activities in motion require a different approach. In 2011, a European consensus determined that no single static posturography test is available with reasonable sensitivity and specificity for the diagnosis of balance disorders, and that perturbation techniques could probably increase the diagnostic yield of posturography [7].

A development to static posturography is computerized dynamic posturography (CDP). CDP enables researchers to separately measure visual and proprioceptive influence on the complex activity of maintaining an upright posture by altering postural and visual input. The quantitative analysis of this measurement is reported as Somatosensory Organization Test (SOT) [8]. Initial CDP systems used an immovable graphic surrounding the patient to influence the visual response, while further studies employed surround displays to introduce more variability into the visual field. A recent development in the CDP and SP is adding head movements to static trials, which might improve the sensitivity and specificity of those methods to identify patients at risk of falls [9]. The most recent advancements in research on the measurement of posture include mobile posturography solutions, which are smaller, more versatile, and have been validated for static and dynamic measurements [10]. Current research in mobile posturography focuses on analyzing posture parameters acquired in a three-dimensional plane in movement during everyday activities or balance-specific clinical tests [11].

Virtual reality is a rapidly developing field in medicine. In 2018, 1634 papers were listed on PubMed under the keyword “virtual reality,” a number which had almost doubled 3 years later, with 2949 papers listed in 2021. Recent advancements in the field of VR have made the devices more portable, lightweight, and capable of recreating a more real-life environment. The concept of VR has changed from an environment surrounded by flat screens or projectors to the development of head-mounted displays. Several devices are available on the market; some require a cable connection to a computer, while others are standalone devices. The cost of this technology has also drastically decreased and is now in the range of 500–2000 USD. A majority of commercially available VR systems include a built-in magnetometer, accelerometer, and gyroscope to allow immersive replication of movement in the virtual surroundings. These same hardware components are the basis of mobile posturography devices. 

The aim of this review is to conduct a literature search on the application of immersive virtual reality delivered using a head-mounted display in posturographic measurements. 

## 2. Materials and Methods

### Literature Search

On 1 March 2022, an extensive search was performed in the PubMed and Scopus electronic databases for papers published in English. The keywords “virtual reality” and “posturography” were used in the search, and there were no time constraints. The Preferred Reporting Items for Systematic Reviews and Meta-analyses (PRISMA) guideline was applied in this review [12]. The criteria for inclusion in this review were: original studies reporting the use of a head-mounted device for virtual reality stimulation and concurrent use of a posturographic system (either static, dynamic, or mobile) for the assessment of balance. Conference proceedings and case studies were excluded.

## 3. Results

**Figure 1:**
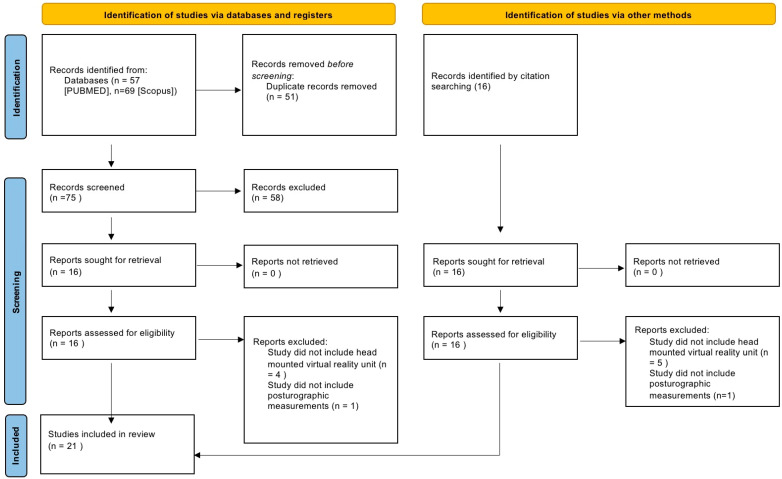
PRISMA 2020 flow diagram for systematic reviews.

Initial screening, after the removal of duplicate entries, included 89 papers. Studies were first identified independently by two of the authors, based on titles and abstracts, for compliance with the inclusion and exclusion criteria. Discrepancies (10%) were discussed until a consensus was reached. Sixteen studies were selected for full-text retrieval; all selected manuscripts were successfully retrieved. Full-text manuscripts were independently screened by two of the authors. An additional 16 records were identified by reference checking. Discrepancies were discussed until a consensus was reached. Finally, 21 studies were included in this review. A PRISMA flowchart detailing the literature selection process is presented in Figure 1. Included manuscripts are summarized in Table 1.

### 3.1. Population of Patients

Eight studies (38%) were exploratory and conducted on a group of healthy individuals (seven studies involved adults, and one study reported the application of VR stimuli in the postural evaluation of children). Thirteen studies (62%) examined populations with peripheral vestibular disorders of different origins: Meniere’s disease (two studies), Benign Paroxysmal Positional Vertigo (three studies), vestibular migraine (one study), or older patients with a history of falls (two studies) related to unspecified vestibular disorders. Among the studies that evaluated the application of VR as an additional visual perturbation in posturography, nine studies (69%) had a control group of healthy volunteers and evaluated the differences between mean posturographic parameters between groups. Of the presented case-control studies, most did not report diagnostic accuracy measures, such as sensitivity or specificity, nor did they attempt to establish cut-off values for the evaluated parameters to differentiate between groups. Only one study, by Tossavainen et al. [24], calculated univariate and multivariate classifiers to evaluate the application of VR and posturography in differentiating between groups of patients with Meniere’s disease and healthy controls. Of the presented parameters, Vertical Ground Force Power Fraction (VFPF) was considered a fairly good classifier, with an accuracy of 68–81%, and it consistently outperformed measures of mean velocity. These results are difficult to compare with other studies included in this review, as this was the only study to measure VFPF. 

### 3.2. Virtual Reality Environments

For the purposes of this review, we have classified the VR environments into three different groups. The first group comprised the majority of the analyzed manuscripts (76%), which defined virtual reality as the projection of relatively simple computer-generated images via head-mounted displays: bars, dots, and cylindrical or tunnel shapes rotating in a two-dimensional plane (flat). We refer to this sort of VR as “optokinetic” perturbation, because of the resemblance to conventional optokinetic trials. The results reported by the authors of the manuscript support this method as an effective way of delivering conflicting visual information and increasing body sway. These studies were also limited by the technology available at the time of publication, as the computing power of processors and the resolution of portable HMDs produced before 2010 were insufficient to produce the vast and dynamic environments present in modern HMDs. 

The second group of VR environments was defined as complex VR. Here, one of the studies used static two-dimensional real-life photography projected onto the displays in the HMD, which interacted with the user’s head movement, giving the sensation of “being” in the environment. The four remaining studies used a complex, dynamic, three-dimensional virtual environment, in which the user could look around and perceive the depth of the image, which is necessary for a fully immersive VR experience. They were among the latest results published and used commercial HMDs available in the last 10 years.

Regarding the equipment used to generate VR, three studies used the Oculus Rift HMD (Meta, California, USA). The Oculus Rift Headset was announced in 2015 and was available for $599. The HMD required a constant PC connection and was capable of a 1080 × 1200 per-eye resolution. The horizontal field of view was 87 degrees, which, when compared to the Virtual Research V8 HMD used by Tossavainen et al. [22,23,24], is an almost two-fold improvement in resolution, a 27-degree wider horizontal field of view, and a refresh rate of 90 Hz (60 Hz is required for the smooth perception of motion). The Oculus Rift was discontinued in 2021 and has been replaced by more capable devices produced by the same manufacturer, with greater resolution and capable of standalone function (i.e., it does not require a constant PC connection), such as the Oculus Quest 2 (Meta, Menio Park, CA, USA).

One study utilized the HTC VIVE (HTC, New Taipei, Taiwan), a computer-powered VR HMD with a per-eye resolution of 1080 × 1200 pixels and a wide horizontal field of view of 108 degrees, which is the widest offered among the devices included in this review. 

Thirteen studies used the Balance Rehabilitation Unit (BRU™). BRU comprises a computer and software, a metal structure, support with loops and a protection belt, a force platform, virtual-reality goggles, an accelerometer, and a foam pillow. The device follows a pre-installed protocol of testing individuals, which consists of 10 different sensorial conditions: (1) open eyes; (2) closed eyes; (3) on the medium density foam pillow and closed eyes; (4) saccadic stimulation; (5) left-to-right horizontal optokinetic stimulation; (6) right-to-left horizontal optokinetic stimulation; (7) top-to-bottom vertical optokinetic stimulation; (8) bottom-to-top vertical optokinetic stimulation; (9) horizontal optokinetic stimulation associated with slow and uniform rotational head movements; and (10) vertical optokinetic stimulation associated with slow and uniform flexion and extension of the head. The virtual reality goggles were used for optokinetic stimulation in conditions 4–10 [25]. None of the manuscripts detailing the use of the BRU detailed the technical specifications of the VR goggles used in this device, and no images from the environment were available. Therefore, based on the description of the sensorial conditions, we evaluated this VR environment as optokinetic stimulation. The device is similar in principle to CDP, where fixed visual reference points were substituted by optokinetic stimulation delivered via VR goggles. The technical specifications of VR headsets used in analyzed manuscripts are summarized in Table 2.

Only one study by Altin et al. [19] describes an additional use of simple audio stimuli in a cognitive exercise, combined with VR; however, the audio was generated externally and did not correspond with the VR environment. None of the studies used or assessed the influence of spatial (surround) audio stimulation in immersive VR on balance. It is worth mentioning that. with the development of modern HMDs, a new opportunity emerges to utilize multi-directional sound stimulation when creating virtual “soundscapes” [33]. The idea of a virtual soundscape is based on the listener’s weighted perception of different sounds present in the environments that are being recreated in VR. Furthermore, spatial sound adds to the perception of “being” in the virtual environment and is nowadays considered a crucial factor in an immersive VR experience [34]. Sound therapy is well-established to address tinnitus, which is a symptom frequently experienced by people with balance disorders, and the inclusion of VR in sound therapy is beneficial to the patient [35]. In future studies on the influence of VR on balance, the influence of spatial sound should be explored.

### 3.3. Posturography Evaluation

One study reports concurrent results of posture measurement using the HMD and a force plate [17]; two other studies mention the capability of tracking the head position during measurement, but do not report the results of such measurements. All of the studies report COP measurements from the force plate, with the majority using COP velocity or sway area as the main output parameter. In comparison, the study by Marchetto et al. [16] analyzed the head position with angular velocity values, as in other studies using body-mounted sensors of posture [36]. The study protocols were different across the manuscripts, as were the VR environments; therefore, a meta-analysis of posturography parameters and VR influence on stability was not attempted in this manuscript. 

## 4. Discussion

Tossavainen et al. were one of the first researchers interested in implementing VR in posturography studies as a method of eliciting additional visual perturbation [22]. In a cycle of publications, the authors have studied the impact of simple yet dynamic visual stimuli delivered via HMD on stability measured by static posturography [22,23,24]. They noted that a dynamic stimulus had a greater impact on increasing body sway parameters measured by COP velocity and sway path. While they noted that the output parameters could be affected by the weight of the HMD (1 kg), this limitation has decreased over time. In comparison, a modern HMD, e.g., the Oculus Quest 2, weighs half of this, at 503 g, with a default resolution of 1832 × 1920 per eye and a refresh rate of 72 Hz [37].

Technological advancements in the field of VR have made the technology far more immersive than it was with simple 3D shapes. The majority of the analyzed manuscripts (76%) report using a very simple, generic virtual environment. Those studies mainly utilized the BRU or were conducted in the early stages of VR research. More recent publications explore the influence of complex VR environments on balance; because of the vast technological improvement in that field, the different studies’ results are difficult to compare. Currently, developers use a wide variety of tools to prepare VR applications. The most commonly used development environments are the Unity and Unreal engines. They allow for the organization and publication of VR projects built on 3D (e.g., 3ds max, Blender) and 2D (e.g., Photoshop, Illustrator) graphics materials under object-oriented C#/C++ codes. In addition, hardware manufacturers and developer communities can integrate applications with specific HMDs.

An important parameter of VR headsets is the field of view (FoV) of the built-in displays. In VR science, an observer’s “natural” FoV of the real world without any head-mounted displays is referred to as the external FoV. The relationship between FoV and the effect of cybersickness in VR is well-known; the greater the FoV of the virtual scene, the more intense the cybersickness symptoms in the exposed individual [38]. An interesting study by van Emmerik et al. [39] explored the difference between the external FoV and the internal FoV (i.e., the field of view of the displayed virtual surrounding), and found that the smaller the difference between the internal and external FoVs, the greater the cybersickness effect. This observation contrasts with the popular opinion that cybersickness arises from the incongruence between the observer’s viewpoint inside and outside the virtual environment. It is important to note that an internal FoV of over 100 degrees is perceived as an ultra-wide angle, otherwise known as the “fish-eye” perspective, which does not seem natural to the observer. Therefore, in medical applications of VR, fields of view of a maximum of 100 degrees seem sufficient for an immersive experience. The FOV alone is not the only parameter that may induce symptoms of cybersickness. This phenomenon is being researched but, thus far, it has not been clearly explained. It has been established that the effects of cybersickness arise with increasing exposure time. However, an adaptation effect is also observed, and the symptoms can gradually alleviate with consistent exposure to VR [40].

In a study from 2020, Wittstein et al. [17] explored the possibility of replacing SOT with dynamic posturography with VR measurements, but they did not use VR as a standalone measure. So far, only one study by Marchetto [16] has used a modern HMD as a standalone device to both deliver the visual stimulus and report posturographic parameters. Two important conclusions can be drawn from that manuscript: head position has a good correlation with COP measurement, and standalone tracking with a VR headset is a possible and plausible alternative to expensive posturography equipment. However, the study sample was relatively small, and studies on a wider population are required to assess the effects of age and gender on head referenced measurements.

Future research should focus on the possibility of using HMDs as standalone devices for both the measurement and delivery of sensorial perturbations. Such technologies may reduce the cost of vestibular testing and increase the diagnostic yield of posturography in vestibular disorders. A recent update on clinical practice guidelines in vestibular hypofunction by Hall et al. [41] underlines the need for exploring the high-end technologies and telehealth in vestibular physical therapy. Further quantitative research into the role of immersive VR on postural stability can lead to effective dosing of VR during vestibular rehabilitation therapy.

## 5. Conclusions

This review of the literature shows that virtual reality has found its use in aiding traditional posturographic evaluation. The use of VR increases postural sway and can be used as an additional sensorial trial. Only one manuscript has evaluated VR HMD as a standalone posturography device and found a good correlation with traditional COP measurement. This line of research should be explored in larger trials.

## Figures and Tables

**Table 1:** Studies included in this review that explore the application of virtual reality to measurements of posture.

Study Authors	Study Size	Study Groups	Study Type	Measure	VR Device	VR Environment	Source of Postural Data
Jurkojć et al., 2017 [13]	23	1. Healthy volunteers	Exploratory	COP velocity COP sway area	Oculus Rift	Complex 3D scenery	Force plate
Cesaroni et al., 2019 [14]	2630	1. Healthy volunteers 2. Vestibular migraine	Cross-sectional	COP sway area COP velocity LOS	BRU VR	Optokinetic	Force plate
Ghiringhelli et al., 2011 [15]	50	1. Healthy volunteers	Exploratory	COP velocity COP sway area LOS	BRU VR	Optokinetic	Force plate
Marchetto et al., 2019 [16]	10	1. Healthy volunteers	Exploratory	COP velocity COP sway area Head angular velocity	Oculus Rift	Complex 3D scenery	1. Force plate 2. HMD
Wittstein et al., 2020 [17]	20	1. Healthy volunteers	Exploratory	COP velocity COP sway area A-P; M-L; SOT VR SOT	HTC VIVE	Complex 3D scenery	Force plate
Gazzola et al. 2019 [18]	76	1. Healthy volunteers 2. Vestibular disorder (unspecified)	Cross-sectional	COP velocity COP sway area LOS	BRU VR	Optokinetic	Force plate
Altin et al., 2020 [19]	30	1. Healthy volunteers	Exploratory	SOT Adaptation Test (ADT)	Oculus Rift	Complex 3D scenery	Force-plate
Macedo et al., 2014 [20]	123	Older people with chronic vestibular dysfunction	Cross-sectional	COP sway area COP velocity	BRU VR	Optokinetic	Force plate
Lee et al., 2004 [21]	30	1. Healthy volunteers (children)	Exploratory	COP sway area, direction	i-glasses SVGA	Complex 2D scenery	Force plate
Tossavainen et al. 2001 [22]	30	1. Healthy volunteers	Exploratory	COP length	Virtual Research V8	Optokinetic	Force plate
Tossavainen et al., 2003 [23]	22	1. Healthy volunteers	Exploratory	COP velocity	Virtual Research V8	Optokinetic	Force plate
Tossavainen et al., 2006 [24]	110	1. Healthy volunteers 2. Meniere’s disease	Cross-sectional	Vertical force power fraction (VFPF) COP velocity	Virtual Research V8	Optokinetic	Force plate
Monteiro et al., 2012 [25]	90	1. Healthy volunteers 2. BPPV	Cross-sectional	COP sway area COP velocity	BRU VR	Optokinetic	Force plate
Kessler et al., 2011 [26]	104	1. Healthy volunteers 2. Multiple sclerosis	Cross-sectional	COP sway area COP velocity	BRU VR	Optokinetic	Force plate
Cusin et al., 2010 [27]	70	1. Healthy volunteers 2. Meniere’s disease	Cross-sectional	COP sway area COP velocity	BRU VR	Optokinetic	Force plate
Duque et al., 2013 [28]	90	Older people with a history of falls	Experimental	COP sway area COP velocity	BRU VR	Optokinetic	Force plate
Alahmari et al., 2014 [29]	90	1. Healthy volunteers 2. Vestibular disorder	Cross-sectional	COP sway area COP velocity	BRU VR	Optokinetic	Force plate
Kasse et al., 2010 [30]	20	1. BPPV	Experimental	COP sway area COP velocity	BRU VR	Optokinetic	Force plate
Lanca et al., 2013 [31]	23	1. BPPV	Experimental	COP sway area COP velocity	BRU VR	Optokinetic	Force plate
Kasse et al., 2010 [30]	66	1. BPPV 2. Healthy volunteers	Experimental	COP sway area COP velocity	BRU VR	Optokinetic	Force plate
Suarez et al., 2006 [32]	26	Older people with a history of falls	Experimental	COP sway area COP velocity	BRU VR	Optokinetic	Force plate

**Table 2:** Technical specifications of the head-mounted displays used in the studies included in this review. Based on specifications available in source manuscripts and producer brochures if available at time of publication.

Model Name	Oculus Rift	HTC VIVE	i-Glasses SVGA	Balance Rehabilitation Unit (BRU)	Virtual Research V8
Manufacturer	Meta, (previously Oculus VR), USA	HTC, Taiwan	Mindflux, USA	Interacoustics	Virtual Research Systems, USA
Resolution per eye	1080 × 1200 pixels	1080 × 1200 pixels	800 × 600	Data unavailable	640 × 480 pixels
Refresh rate	90 Hz	90 Hz	60–100 Hz	Data unavailable	60 Hz
Field of view	87° horizontal 88° vertical	108° horizontal 97° vertical	26° diagonal	Data unavailable	60° diagonal
Weight	470 g	470 g	200 g	Data unavailable	1000 g
Tracking system	6 DOF built-in	6 DOF marker-based	Optional head-worn tracker	Not implemented	Not implemented
Announced	6 May 2015	1 March 2015	2004	2010	1998
Cost at introduction	599 USD	799 USD	Data unavailable	Data unavailable	Data unavailable

## Data Availability

The data presented in this study are available in the original articles listed in the references below.

## Abbreviations

The following abbreviations are used in this manuscript:

VR	Virtual reality
HMD	Head-mounted display
SP	Static posturography
COP	Center of pressure
CDP	Computerized dynamic posturography
SOT	Sensory organization test
LOS	Limits of stability
A-P	Anteroposterior displacement in force-plate posturography
M-L	Mediolateral displacement in force-plate posturography
BRU	Balance rehabilitation unit
BPPV	Benign positional paroxysmal vertigo
3D	Three-dimensional virtual environment
2D	Flat, two-dimensional virtual environment
DOF	Degrees of freedom
